# Diet Quality and Subsequent Incidence of Upper Gastrointestinal Cancers: Results from the Golestan Cohort Study

**DOI:** 10.34172/aim.2023.74

**Published:** 2023-09-01

**Authors:** Majid Namaki, Maryam Hashemian, Abbas Arj, Hossein Poustchi, Gholamreza Roshandel, Amir Hossein Loghman, Sadaf G. Sepanlou, Akram Pourshams, Masoud Khoshnia, Abdolsamad Gharavi, Nafiseh Abdolahi, Sima Besharat, Azita Hekmatdoost, Paul Brennan, Sanford M. Dawsey, Farin Kamangar, Paolo Boffetta, Christian C. Abnet, Reza Malekzadeh, Mahdi Sheikh

**Affiliations:** ^1^Department of Internal Medicine, Faculty of Medicine, Kashan University of Medical Sciences, Kashan, Iran; ^2^Department of Biology, School of Art and Sciences, Utica College, Utica, New York, USA; ^3^Liver and Pancreatobiliary Diseases Research Center, Digestive Diseases Research Institute, Tehran University of Medical Sciences, Tehran, Iran; ^4^Digestive Oncology Research Center, Digestive Diseases Research Institute, Tehran University of Medical Sciences, Tehran, Iran; ^5^Golestan Research Center of Gastroenterology and Hepatology, Golestan University of Medical Sciences, Gorgan, Iran; ^6^Digestive Disease Research Center, Digestive Diseases Research Institute, Tehran University of Medical Sciences, Tehran, Iran; ^7^Golestan Rheumatology Research Center, Golestan University of Medical Sciences, Gorgan, Iran; ^8^Departments of Clinical Nutrition and Dietetics, Faculty of Nutrition and Food Technology, National Nutrition and Food Technology Research Institute, Shahid Beheshti University of Medical Sciences, Tehran, Iran; ^9^Genomic Epidemiology Branch, International Agency for Research on Cancer (IARC/WHO), Lyon, France; ^10^Metabolic Epidemiology Branch, Division of Cancer Epidemiology and Genetics, National Cancer Institute, Bethesda, MD, United States; ^11^Department of Biology, School of Computer, Mathematical, and Natural Sciences, Morgan State University, Baltimore, MD, United States; ^12^Tisch Cancer Institute, Icahn School of Medicine at Mount Sinai, New York, NY, United States

**Keywords:** Diet, Digestive, Epidemiology, Malignancies, Nutrition

## Abstract

**Background:**

Recent evidence suggests overall diet quality, as assessed by dietary scores, may play a role in the development of upper gastrointestinal (UGI) cancers. However, the existing dietary scores are derived from high-income countries with different dietary habits than regions with the highest burden of UGI cancers, where limited data is available. This study aimed to investigate the association between overall diet quality and risk of esophageal and stomach cancers in a high-risk region for UGI cancers.

**Methods:**

We recruited 50045 individuals aged 40-75 between 2004-2008 from northeastern Iran and followed them annually through July 2020. Data on demographics, diet, and various exposures were collected using validated questionnaires. Diet quality was assessed by calculating the Healthy Eating Index (HEI), Alternative Healthy Eating Index (AHEI), Alternative Mediterranean Diet (AMED), Dietary Approaches to Stop Hypertension (DASH), and World Cancer Research Fund–American Institute for Cancer Research (WCRF-AICR) scores.

**Results:**

During an average 12 years of follow-up, 359 participants developed esophageal cancer and 358 developed stomach cancer. After adjustments, each standard deviation increase in baseline dietary scores was associated with up to 12% reduction in esophageal cancer risk and up to 17% reduction in stomach cancer risk. Esophageal cancer showed stronger inverse associations with adherence to AMED (HR_Q4-vs-Q1_=0.69 (0.49–0.98), *P*-trend=0.038). Stomach cancer showed stronger inverse correlation with WCRF-AICR (HR_Q4-vs-Q1_=0.58 (0.41–0.83), *P*-trend=0.004), and DASH (HR_C4-vs-C1_=0.72 (0.54–0.96), *P*-trend=0.041). These associations were comparable across different population subgroups. We did not observe significant associations between HEI and AHEI scores and UGI cancers in this population.

**Conclusion:**

Despite the differences in consuming individual food groups, adherence to the available dietary recommendations (derived from high-income countries) was associated with lower risk for subsequent esophageal and gastric cancers in this high-risk population. Educating the public to have a healthy eating pattern might be an effective strategy towards prevention of UGI cancers in high-risk regions.

## Introduction

 In 2020, an estimated 1.7 million individuals were diagnosed with upper gastrointestinal (UGI) cancers and 1.3 million died from these cancers.^1^ The burden of these cancers is highest in low- and middle- income countries (LMICs), where around 85% of UGI cancer deaths occur.^1,2^ Between 30%‒50% of cancer cases are preventable through lifestyle modification.^3,4^ Given that diet constitutes a major lifestyle element, developing nutritional policies has emerged as an international public health priority to protect against cancer and other non-communicable diseases.^4–6^

 Recent evidence indicates that whole diet quality (assessed by dietary scores) might have an important role in the development of UGI cancers.^4,7,8^ However, the strength and direction of dietary associations with UGI cancers seem to be organ site-specific,^8^ and discrepancies have been observed for these associations across geographic regions.^7^ Furthermore, the available dietary scores were derived from high-income countries (HICs) where dietary habits are different from regions with the highest burden of UGI cancers.^9^ However, limited or no data is available on whole diet quality and risk of UGI cancers from high-risk regions.^4,7^ Therefore, rigorous research in LMICs is needed to investigate the applicability of the available dietary recommendations for reducing the risk of UGI cancers and informing cancer prevention policies in these high-risk regions.^4,8,10^

 Northeastern Iran has long been known as a high-risk region for UGI cancers.^11^ Decades of epidemiologic studies suggest dietary factors might contribute to the risk of UGI cancers in this region.^11–14^ However, most studies only focused on intake of specific food groups and nutrients rather than assessing whole diet quality.^12–19^ Based on 571 042 person-years of follow-up within the Golestan Cohort Study (GCS) in northeastern Iran, we assessed baseline diet quality in this population using five dietary scores (Healthy Eating Index 2015 [HEI], Alternative Healthy Eating Index 2010 [AHEI], Alternative Mediterranean Diet [AMED], Dietary Approaches to Stop Hypertension [DASH], and World Cancer Research Fund–American Institute for Cancer Research [WCRF-AICR]) and investigated whether baseline diet quality was associated with the risk of developing UGI cancers in this population.

## Materials and Methods

###  Study Population and Design

 The GCS is a prospective population-based cohort of 40‒75-year-old individuals living in the Golestan province in northeastern Iran. The details of the GCS design have been published previously.^20,21^ After the pilot phase, 50,045 people were recruited to the GCS between 2004 and 2008, from 326 urban and rural areas of Gonbad, Maraveh-tappeh, Kalaleh, and Aq-qala districts of the province.^21^ Participants who had been diagnosed with upper GI cancers before enrollment, those who were unwilling to participate, and temporary residents were excluded from the GCS. For this analysis, we also excluded participants who did not have a fully completed Food Frequency Questionnaire (FFQ) and those with extreme energy intake. All participants provided a written informed consent before enrollment in the study. The GCS was approved by the institutional review boards of the Digestive Disease Research Institute of Tehran University of Medical Sciences, the International Agency for Research on Cancer, and the US National Cancer Institute.

###  Questionnaires and Data Collection

 At enrollment, the participants were interviewed by trained staff to complete two validated questionnaires^20-22^; a general questionnaire to collect data on demographics, lifestyle, medical history, socioeconomic status, and various exposures; and an FFQ that was developed by a team of Iranian nutritionists and contained detailed inquiries about the frequency and amount of consuming 116 food items.^22^

 Participants were asked about ever regular use of opium (a widely used addictive narcotic), tobacco, and alcohol, and if applicable, the duration, frequency and consumption amount of each substance. We calculated the cumulative opium used in *nokhod*-years (*nokhod* is a local unit for opium ≈ 0.2 g), and cumulative cigarettes smoked in pack-years (pack = 20 cigarettes), by calculating the number of units consumed per day multiplied by the number of consumption years. Since alcohol consumption is rare in this region, we categorized the participants based on ever/never regular alcohol consumption.

 To evaluate socioeconomic status, we used the quartiles of a composite wealth score that was created previously using multiple correspondence analysis on the following variables: property ownership, structure and size of the house, vehicle ownership, and having a television, refrigerator, freezer, vacuum cleaner, or washing machine at home.^23^ The participants were also asked about having any formal education and if applicable, years of education.

 The GCS included mostly rural populations whose main daily physical activity was related to their activity at work. The participants were asked whether they worked every month throughout the year and if their work type included intense physical activity. Accordingly, they were categorized based on their occupational activity as “irregular non-intense”, “regular non-intense”, and “intense” physical activity.^24^ The participants also underwent a brief physical examination and anthropometric measurements. Body mass index (BMI) was calculated by dividing the participants’ weight by their squared height (kg/m^2^).

###  Dietary Scores and Energy Intake

 We previously showed that the FFQ provides valid and reliable measurements of intake for energy, and various food groups and nutrients in this population.^22^ A brief description of the questionnaires’ validation studies is provided in Supplementary file 1 (File S1). Using the FFQ, we calculated the HEI, AHEI, AMED, DASH, and WCRF/AICR scores to quantify baseline diet quality in this population.^9^ Details for calculating the dietary scores are presented in Supplementary file 1 (File S1, Tables S1 and S2). Briefly, the Iranian food tables were used to extract the energy and nutrient contents.^25^ When the nutrient contents were not available in the Iranian tables, the United States Department of Agriculture reports (release 23) were used.^26^ The Food Patterns Equivalents Database (FPED) 2013‒14 was used to convert the daily intakes from grams to cup and ounce equivalents to create components of the HEI and AHEI scores.^27^

 The HEI score evaluates adherence to the dietary guidelines for Americans and has 13 components for a total of 100 points.^28^ The energy density model was used to calculate the components per 1000 kcal/d. The AHEI score evaluates adherence to the dietary guideline created by Harvard University and has 11 components for a total of 110 points.^29^ The AMED score evaluates adherence to the Mediterranean diet and includes nine components for a total of 9 points and uses sex-specific median of the participants’ intake for scoring.^30^ The DASH score evaluates adherence to DASH diet for preventing hypertension that was created by Fung et aland includes 8 components for a total of 40 points and uses sex-specific quintiles of the participants’ intake for scoring.^31^ The WCRF/AICR score evaluates adherence to the recommendations by WCRF to prevent cancer and includes 7 dietary components and 3 non-dietary components. To allow comparability with the other scores, only the dietary components were included in this study.^32^ The participants received 0, 0.5, or 1 point if they did not meet the recommendation, met an intermediate recommendation, or met the recommendations, respectively, for a total of 7 points.^33^ All participants received a zero for alcohol or whole grain in the dietary scores including these two items because alcohol drinking or whole grain consumption are not common in this population.

###  Follow-up and Outcome Ascertainment

 Around 99% of the participants have been successfully followed since enrollment using annual telephone surveys. In case of reporting incident cancers, a staff visits the patient’s home to complete a detailed questionnaire. Then, a team visits the corresponding medical centers to gather copies of relevant medical documents, which are then reviewed by 2-3 expert physicians to verify the diagnosis of cancer. Additionally, the cohort records are matched to the Golestan Population-based Cancer Registry database to minimize misclassifications in the diagnosis of cancer. The final diagnosis of cancers are recorded based on the 10th revision of the International Statistical Classification of Diseases and Related Health Problems (ICD-10).^34^ Incident cancers with ICD-10 codes C15 (esophageal cancer) and C16 (stomach cancer) were the outcomes of interest for this analysis. Given that around 90% of esophageal cancer cases were squamous cell carcinoma, we did not separate this outcome by histologic subtypes.

###  Statistical Analyses

 After checking the proportional hazard assumptions, Cox proportional hazards models were applied to estimate hazard ratios (HRs) and 95% confidence intervals (CIs) for the association between dietary scores and risk of UGI cancers. For each participant, age at recruitment was defined as the entry time, while the exit time was defined as age at the time of UGI cancer diagnosis, death, or last contact (through July 18, 2020), whichever came first. The models were adjusted for sex, residence district, socioeconomic status, ethnicity, education, BMI, physical activity level, pack-years of cigarettes smoked, *nokhod*-years of opium used, ever alcohol consumption, and energy intake (kcal/d).

 To evaluate the association of dietary scores with risk of UGI cancers, we used two approaches: first, we divided the dietary scores by their standard deviations (SD) and treated them as continuous variables in the models to assess the risk of UGI cancers associated with each increment in the SD of the scores. Then, for each dietary score, we categorized the participants into sex-specific quartiles and treated the lowest quartile (worst diet quality) of each dietary score as the reference category and compared the risk of UGI cancers across the other quartiles. For the latter analysis, we also calculated the P values for trend by assigning consecutive integers to consecutive quartiles.

 Sensitivity analyses were performed after dropping the first two years of follow-up and after excluding cancer cases who did not have histologic confirmation. Furthermore, for better interpretation, we stratified the analysis by sex (male/female), wealth score (lower/higher than median), and BMI ( ≤ 25/ > 25). The Stata statistical software version 16 (StataCorp., College Station, TX, USA) was used for all statistical analyses. All reported *P* values were two-tailed, and *P* < 0.05 was considered as statistically significant.

## Results

###  Baseline Demographics & Dietary Patterns

 A total of 50 045 individuals were recruited to the GCS, of whom 1562 were excluded because of having reported a diagnosis of GI cancers at enrolment (n = 29), having ≥ 30 missing responses on the baseline FFQ (n = 996); and having a calculated energy intake of more than twice the interquartile range above the 75th percentile (3690 kcal/d for women and 4145 kcal/d for men) or less than twice the interquartile range below the 25th percentile of energy intake (300 kcal/d for women and 525 kcal/d for men) (n = 537). The remaining 48,483 participants were included in this analysis.

 The median follow-up time was 12.0 years (interquartile range: 11.1–13.2 years). At follow-up, 359 participants were diagnosed with esophageal cancer and 358 were diagnosed with stomach cancer. Of the total UGI cancer cases, 587 (82%) had histological confirmation and the remaining 130 (18%) were diagnosed by verbal autopsy and other medical records. Participants with better baseline diet (higher dietary scores) had higher BMI and wealth score; were more likely to be educated, to live in urban areas, and to drink alcohol; and were less likely to be Turkmen and use opium (Table 1).

**Table 1:** Baseline Characteristics of Participants in the Golestan Cohort Study for the Total Population and Participants in the Lowest and Highest Quartiles of Assessed Dietary Scores

**Scores (Median/Maximum Possible Score)**	**Total Population**	**HEI (34/100)**		**AHEI (40/110)**		**AMED (3/9)**		**DASH (22/40)**		**WCRF-AICR (2.5/7)**	
		**Q1**	**Q4**	**Q1**	**Q4**	**Q1**	**Q4**	**Q1**	**Q4**	**Q1**	**Q4**
Score range for males	—	7–28	41–78	12–35	46–78	0–2	5–7	8–19	24–36	0.5–2	3.5–5.5
Score range for females	—	7–28	41–82	14–35	47–78	0–2	5–7	11–19	24–36	0.5–2	3.5–5.5
Participants ^a^	48483 (100.0)	13279 (27.3)	11491 (23.7)	12644 (26.0)	11269 (23.2)	13480 (27.8)	13216 (27.2)	11609 (23.9)	15851 (32.6)	13939 (28.7)	12311 (25.3)
UGI cancers ^a^	717 (1.4)	249 (1.8)	133 (1.1)	233 (1.8)	136 (1.2)	291 (2.1)	127 (0.9)	215 (1.8)	192 (1.2)	244 (1.7)	145 (1.1)
Age ^b^, years	52.0 ± 8.9	52.0 ± 8.8	52.5 ± 9.0	51.6 ± 8.7	52.9 ± 9.2	53.4 ± 9.2	50.6 ± 8.3	51.6 ± 8.7	52.4 ± 9.0	52.7 ± 9.2	51.6 ± 8.6
BMI ^b^, kg/m^2^	26.6 ± 5.4	25.4 ± 5.2	28.0 ± 5.4	26.1 ± 5.3	27.4 ± 5.4	25.5 ± 5.3	27.9 ± 5.2	26.0 ± 5.4	27.3 ± 5.3	25.6 ± 5.4	27.7 ± 5.2
Gender ^a^											
Male	20503 (42.2)	5799 (43.6)	4798 (41.7)	5505 (43.5)	5032 (44.6)	5677 (42.1)	5553 (42.0)	4940 (42.5)	6762 (42.6)	4744 (34.0)	6473 (52.5)
Female	27980 (57.7)	7480 (56.3)	6693 (58.2)	7139 (56.4)	6237 (55.3)	7803 (57.8)	7663 (57.9)	6669 (57.4)	9089 (57.3)	9195 (65.9)	5838 (47.4)
Place of residence ^a^											
Gonbad urban	9833 (20.2)	1267 (9.5)	4397 (38.2)	1542 (12.2)	3900 (34.6)	1322 (9.8)	4683 (35.4)	1138 (9.8)	5436 (34.2)	1512 (10.8)	4608 (37.4)
Gonbad rural	19 172 (39.5)	6106 (45.9)	3552 (30.9)	5553 (43.9)	3746 (33.2)	6413 (47.5)	4094 (30.9)	5122 (44.1)	5308 (33.4)	6661 (47.7)	3539 (28.7)
Kalaleh	10 605 (21.8)	3387 (25.5)	1670 (14.5)	2906 (22.9)	1881 (16.6)	3367 (24.9)	2141 (16.2)	2857 (24.6)	2683 (16.9)	3441 (24.6)	2230 (18.1)
Aq-qala	5530 (11.4)	1503 (11.3)	1243 (10.8)	1396 (11.0)	1311 (11.6)	1131 (8.3)	1764 (13.3)	1609 (13.8)	1449 (9.1)	1384 (9.9)	1099 (8.9)
Maraveh-tappeh	3343 (6.9)	1016 (7.6)	629 (5.4)	1247 (9.8)	431 (3.8)	1247 (9.2)	534 (4.0)	883 (7.6)	975 (6.1)	941 (6.7)	835 (6.7)
Ethnicity ^a^											
Turkmen	35 845 (73.9)	10 004 (75.3)	7711 (67.1)	10 636 (84.1)	6978 (61.2)	10 719 (79.5)	8884 (67.2)	9260 (79.7)	10 540 (66.4)	10 356 (74.3)	8450 (68.6)
Education ^a^											
Illiterate	33 984 (70.0)	10 550 (79.4)	6463 (56.2)	9595 (75.8)	6621 (58.7)	11065 (82.0)	7218 (54.6)	8963 (77.2)	9611 (60.6)	11 446 (82.1)	6608 (53.6)
Wealth score ^a^											
Highest quartile	12 135 (25.0)	1411 (10.6)	5248 (45.6)	2236 (17.6)	4375 (38.8)	1539 (11.4)	5701 (43.1)	1663 (14.3)	5823 (36.7)	1526 (10.9)	5333 (43.3)
Physical activity ^a^											
Intense	5623 (11.6)	2063 (15.5)	842 (7.3)	1548 (12.2)	1103 (9.7)	1560 (11.5)	1295 (9.8)	1657 (14.2)	1518 (9.5)	1490 (10.6)	1408 (11.4)
Tobacco											
Ever user^a^	8353 (17.2)	2538 (19.1)	1882 (16.3)	2249 (17.7)	1975 (17.5)	2309 (17.1)	2215 (16.7)	2091 (18.0)	2655 (16.7)	2117 (15.1)	2489 (20.2)
Pack-years ^bc^	17.1 ± 18.6	18.3 ± 20.2	16.2 ± 17.8	18.0 ± 19.6	16.6 ± 17.4	18.3 ± 20.1	16.0 ± 17.8	17.3 ± 18.9	16.5 ± 17.4	17.9 ± 20.6	16.7 ± 17.4
Opium											
Ever user ^a^	8199 (16.9)	2772 (20.8)	1546 (13.4)	2253 (17.8)	1678(14.8)	2609 (19.3)	1748 (13.2)	2095 (18.0)	2358 (14.8)	2735 (19.6)	1845 (14.9)
*Nokhod*-years ^b^	57.0 ± 109.0	63.6 ± 105.2	50.5 ± 121.7	65.4 ± 136.1	50.8 ± 91.5	59.5 ± 120.0	51.7 ± 101.5	60.3 ± 111.6	51.8 ± 93.3	56.2 ± 100.7	57.8 ± 111.7
Alcohol drinking ^a^											
Ever drinker ^a^	1657 (3.4)	305 (2.3)	638 (5.5)	326 (2.5)	615 (5.4)	263 (1.9)	706 (5.3)	291 (2.5)	733 (4.6)	262 (1.8)	745 (6.0)

 We observed significant correlations between the assessed dietary scores (Table 2). DASH and AHEI scores had the highest correlation (r = 0.75), while WCRF-AICR and AHEI had the lowest correlation (r = 0.37) (Table 2). Table 3 illustrates the median daily intake of selected dietary components among the study population. Participants in the highest quartiles of the dietary scores had higher daily intake of total energy, fruits, vegetables, dairy products, and white meat, and had a higher ratio for unsaturated/saturated fatty acid intake. Daily intakes of grains and red meat were not consistent across the quartiles of the assessed scores, and processed meat was rarely consumed in this population (Table 3).

**Table 2:** Pearson’s Correlation Coefficients for the Correlation between the Assessed Dietary Scores

	**HEI**	**AHEI**	**AMED**	**DASH**	**WCRF-AICR**
HEI	1				
AHEI	0.68	1			
AMED	0.54	0.62	1		
DASH	0.57	0.75	0.56	1	
WCRF-AICR	0.40	0.37	0.54	0.43	1

HEI, Healthy Eating Index 2015; AHEI, Alternative Healthy Eating Index 2010; AMED, Alternate Mediterranean Diet; DASH, Dietary Approaches to Stop Hypertension; WCRF/AICR, World Cancer Research Fund/American Institute for Cancer Research index. *P* values for all correlations were < 0.001.

**Table 3:** Selected Dietary Components Assessed by Baseline Food Frequency Questionnaire of the Golestan Cohort Study for the Total Population and Participants in the Lowest and Highest Quartiles of the Assessed Dietary Scores

**Scores(Median/MaximumScore)**	**Total** **Population**	**HEI(34/100)**		**AHEI(40/110)**		**AMED(3/9)**		**DASH(22/40)**		**WCRF-AICR(2.5/7)**	
		**Q1**	**Q4**	**Q1**	**Q4**	**Q1**	**Q4**	**Q1**	**Q4**	**Q1**	**Q4**
Energy, kcal/d ^a^	2122.1 (1772.0–2486.2)	2057.7 (1734.3 –2390.6)	2146.4 (1763.8 –2546.5)	2084.8 (1763.3 –2421.5)	2172.7 (1801.2–2545.5)	1881.4 (1541.2–2206.7)	2350.8 (2019.3–2707.3)	2079.1 (1747.6–2424.6)	2179.1 (1820.2–2544.0)	1789.9 (1469.7–2085.7)	2497.3 (2159.9–2841.5)
Fruits (g/d) ^a^	120.5 (71.6–194.3)	69.2 (41.7–105.2)	197.6 (130.2–295.6)	90.5 (55.8–138.8)	173.0 (109.9–271.6)	72.9 (44.1 –106.0)	187.5 (137.3 –273.4)	77.5 (47.8–115.7)	177.9 (117.3–266.8)	68.5 (42.9–98.2)	238.4 (158.1 –342.4)
Vegetables (g/d) ^a^	113.7 (77.2–160.5)	86.7 (60.8–119.3)	158.0 (111.4–223.0)	90.0 (63.4 - 123.0)	155.2 (108.9–218.0)	77.4 (54.6–100.1)	165.7 (131.1 –220.2)	79.7 (56.9–108.0)	156.6 (115.7–211.0)	77.0 (55.0–102.4)	178.1 (130.5 –241.0)
Grains (g/d) ^a^	430 (319.7–500.4)	444.9 (336.1 –504.0)	377.6 (277.6–485.5)	434.7 (330.3 - 506.5)	407.6 (310.1–490.6)	389.6 (303.3 –479.2)	447.7 (339.5 –514.3)	447.2 (336.1 –511.8)	407.9 (309.9–493.2)	342.5 (246.5 –450.2)	485.6 (386.8 –558.3)
Dairy (g/d) ^a^	166.2 (95.1–259.0)	108.3 (58.2–180.6)	232.7 (155.6–335.4)	149.1 (86.9 - 235.0)	200.3 (119.5–303.2)	124.5 (64.9–210.0)	215.1 (142.9 –310.1)	111.6 (61.6–181.3)	224.9 (151.4–322.5)	117.3 (64.0–192.5)	228.5 (150.8 –334.5)
Red meat (g/d) ^a^	11.4 (5.6–20.7)	8.5 (4.1–15.7)	14.4 (6.9–25.2)	14.9 (8.1–25.2)	9.0 (4.2–16.9)	10.7 (4.5–18.9)	12.3 (6.8–23.0)	13.3 (6.7–22.9)	10.3 (5.0–18.9)	8.3 (3.8–15.7)	15.8 (8.1–27.0)
White meat (g/d) ^a^	54.8 (30.3–87.9)	40.0 (20.8–64.5)	76.7 (45.9–116.8)	35.9 (18.1–58.6)	82.0 (52.3–121.8)	39.9 (19.7–66.3)	71.6 (45.3–107.5)	47.7 (24.9–78.8)	63.1 (36.9–98.6)	46.7 (24.9–76.5)	63.3 (35.9–98.9)
(PUFA + MUFA) / SFA	0.6 (0.5–0.8)	0.6 (0.5–0.7)	0.9 (0.6–1.6)	0.5 (0.4–0.6)	0.9 (0.7–1.3)	0.6 (0.5–0.7)	0.8 (0.6–1.0)	0.6 (0.5–0.8)	0.7 (0.5–0.9)	0.6 (0.5–0.8)	0.7 (0.6–0.9)

HEI, Healthy Eating Index 2015; AHEI, Alternative Healthy Eating Index 2010; AMED, Alternate Mediterranean Diet; DASH, Dietary Approaches to Stop Hypertension; WCRF/AICR, World Cancer Research Fund/American Institute for Cancer Research index; Q1, lowest quartile (worst category of the dietary score); Q4, highest quartile (best category of the dietary score); PUFA, Polyunsaturated Fatty Acids; MUFA, Monounsaturated Fatty Acid, SFA, Saturated Fatty Acid. ^a^Data are presented as median (interquartile range).

###  Adherence to Dietary Scores and Risk of Esophageal Cancer

**Figure 1:**
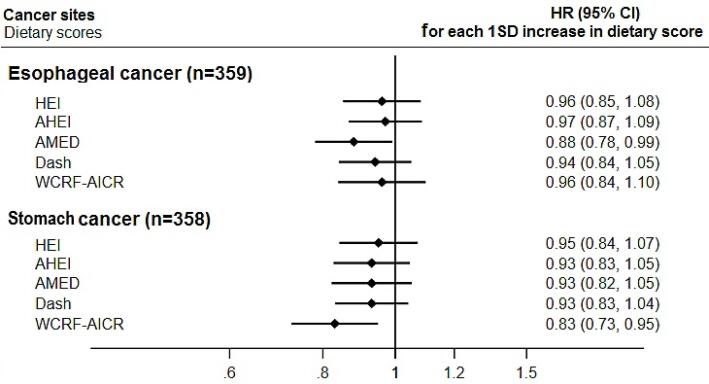
Association between Each Increment in the Standard Deviation (SD) of the Dietary Scores and Risk of Esophageal and Stomach Cancer. The five assessed scores are Healthy Eating Index 2015 (HEI); Alternative Healthy Eating Index 2010 (AHEI); Alternate Mediterranean Diet (AMED); Dietary Approaches to Stop Hypertension (DASH); and World Cancer Research Fund/American Institute for Cancer Research index (WCRF/AICR).

 Among the assessed dietary scores, AMED showed stronger association with esophageal cancer. Participants with the highest vs. lowest AMED score had 31% lower risk for developing esophageal cancer (HR_Q4-vs-Q1_ = 0.69, 95% CI = 0.49–0.98, *P*_trend_ = 0.038) (Table 4). Each increment in the SD of the AMED score was associated with 12% reduced risk for esophageal cancer (Figure 1). The observed estimates for AMED and esophageal cancer risk were attenuated in some of the sensitivity and stratified analyses. However, the trends and point estimates were comparable across males and females (Table S3), participants with lower and higher wealth scores (Table S4), and participant with BMI ≤ 25 and > 25 (Table S5). Further, the results remained similar after excluding cancer cases without histologic confirmation (Table S6), and after dropping the first two years of follow-up (Tables S7).

**Table 4:** Association between Different Dietary Scores and Incidence of Upper Gastrointestinal Cancers in the Golestan Cohort Study

	**Q1**	**Q2**	**Q3**	**Q4**	**Trend *P* Value**
**Esophageal Cancer (n=359)**					
HEI					
No. of participants	13,279	12,032	11,681	11,491	-
HR (95% CI)^a^	1.00	0.86 (0.65–1.13)	0.88 (0.66–1.17)	0.91 (0.66–1.24)	0.48
AHEI					
No. of participants	12,644	12,247	12,323	11,269	-
HR (95% CI)^a^	1.00	0.91 (0.69–1.19)	0.76 (0.56–1.01)	0.92 (0.67–1.25)	0.27
AMED					
No. of participants	13,480	11,151	10,636	13,216	-
HR (95% CI)^a^	1.00	0.71 (0.54–0.94)	0.81 (0.60–1.09)	0.69 (0.49–0.98)	0.038
DASH					
No. of participants	11,609	10,303	10,693	15,851	-
HR (95% CI)^a^	1.00	0.88 (0.66–1.18)	0.76 (0.56–1.03)	0.84 (0.63–1.11)	0.16
WCRF-AICR					
No. of participants	13,939	12,007	10,226	12,311	-
HR (95% CI)^a^	1.00	1.08 (0.82–1.42)	0.93 (0.67–1.30)	0.99 (0.70–1.40)	0.82
**Stomach Cancer (n=358)**					
HEI					
No. of participants	13,279	12,032	11,681	11,491	-
HR (95% CI)^a^	1.00	0.81 (0.60–1.08)	0.99 (0.75–1.30)	0.90 (0.66–1.24)	0.77
AHEI					
No. of participants	12,644	12,247	12,323	11,269	-
HR (95% CI)^a^	1.00	0.89 (0.67–1.18)	0.94 (0.71–1.25)	0.81 (0.59–1.11)	0.28
AMED					
No. of participants	13,480	11,151	10,636	13,216	-
HR (95% CI)^a^	1.00	0.83 (0.63–1.11)	0.89 (0.65–1.20)	0.91 (0.65–1.25)	0.55
DASH					
N of participants	11,609	10,303	10,693	15,851	-
HR (95% CI)^a^	1.00	0.81 (0.60–1.08)	0.84 (0.63–1.13)	0.72 (0.54–0.96)	0.041
WCRF-AICR					
No. of participants	13,939	12,007	10,226	12,311	-
HR (95% CI)^a^	1.00	0.83 (0.63–1.10)	0.76 (0.56–1.05)	0.58 (0.41–0.83)	0.004

HEI, Healthy Eating Index 2015; AHEI, Alternative Healthy Eating Index 2010; AMED, Alternate Mediterranean Diet; DASH, Dietary Approaches to Stop Hypertension; WCRF/AICR, World Cancer Research Fund/American Institute for Cancer Research index ^a^ Models are adjusted for sex, residence district, socioeconomic status, ethnicity, education, BMI, physical activity level, cumulative cigarettes smoked, cumulative opium consumed, alcohol consumption, and energy intake.

###  Adherence to Dietary Scores and Risk of Stomach Cancer

 Among the assessed dietary scores, WCRF-AICR showed the strongest and most consistent association with stomach cancer. Also, better adherence to DASH dietary patterns was associated with lower risk for stomach cancer. Participants with the highest vs. lowest WCRF-AICR score had 42% lower risk (HR_Q4-vs-Q1_ = 0.58, 95% CI = 0.41–0.83, *P*_trend_ = 0.004), and those with the highest vs. lowest DASH score had 28% lower risk (HR_Q4-vs-Q1_ = 0.72, 95% CI = 0.54–0.96, *P*_trend_ = 0.041) for developing stomach cancer (Table 4). Each increment in the SD of the WCRF-AICR score was associated with 17% reduced risk for stomach cancer (Figure 1). The observed estimates were comparable across males and females (Table S3), participants with lower and higher wealth scores (Table S4), and participant with BMI ≤ 25 and > 25 (Table S5). The results remained similar after excluding cancer cases without histologic confirmation (Table S6), and after dropping the first two years of follow-up (Tables S7).

## Discussion

 Analyzing data from over 48 000 participants of the GCS who were followed for a median of 12 years showed an inverse association between baseline diet quality and subsequent risk of UGI cancers. Although the patterns and amount of consuming different dietary groups in this population varied from those reported in Western populations where dietary scores were derived,^35-37^ better adherence to AMED, WCRF-AICR, and DASH dietary recommendations was associated with lower risk for developing esophageal and gastric cancers in this high-risk population. HEI and AHEI scores did not show strong associations with UGI cancers in this population.

 Northeastern Iran has long been known for having one of the highest reported rates for esophageal cancer worldwide.^38^ Decades of epidemiologic research in this area and other high-risk regions have indicated that poor diet may play a role in the etiology of this disease.^11–14,38,39^ However, rather than assessing the overall diet quality, most of these studies focused on the intake of specific dietary factors, nutrients, and food groups. Two interventional studies in a high-risk region of China assessed the effects of multivitamin and micronutrient supplementation in the prevention of esophageal cancer in patients with esophageal squamous dysplasia,^40^ and in the general population.^41^ The results of these two studies showed no to minimum benefit for these interventions in terms of reducing esophageal cancer risk.^42,43^ A meta-analysis of interventional trials also indicated no effects for vitamin and antioxidant supplements in preventing esophageal cancer.^44^ These results and the fact that different dietary factors are consumed in combination, and they usually correlate and interact one with another, highlight the need to consider the overall diet quality in assessing the relationship between diet and risk of UGI cancers.

 In this high-risk population, we utilized five dietary scores to assess the overall diet quality and risk of esophageal and stomach cancers. We found an inverse association between esophageal cancer and adherence to AMED dietary pattern. Adherence to the Mediterranean diet that has a plant-based food foundation has been frequently shown to be associated with reduced risk for esophageal cancer (particularly the squamous cell carcinoma subtype (ESCC)) in HICs.^7,8,45^ On the other hand, greater adherence to the WCRF-AICR dietary recommendation in this population was associated with lower risk for stomach cancer, which was similar to the results from three cohort studies in Europe.^33,46^ We also found greater adherence to DASH dietary pattern to be associated with lower risk for developing stomach cancer. Greater adherence to DASH dietary pattern in this population has been also linked to lower risk of cancer death,^9^ and lower risk of death from gastrointestinal cancers.^47^ Similarly, a recent case-control study from Iran ^48^, a meta-analysis of case-control and cohort studies in the United States and Canada,^49^ and a Markov cohort model study from the United States,^50^ showed a lower risk for stomach cancer with greater adherence to DASH dietary pattern.

 We did not find strong associations between adherence to HEI and AHEI dietary recommendations and risk of UGI cancers in this population. This might be due to using absolute cutoffs for assigning values for consuming dietary components in these indices, which were derived from dietary guidelines for the Americans.^28,29^ Our results showed that the dietary pattern in this population is largely different from high-income countries. For example, in comparison to the EPIC study that includes general populations from 10 European countries,^35–37^ the Golestan population had higher intake of grains and lower intake of red and processed meat, alcohol, dairy products, fruits, vegetables, and unsaturated lipids. These differences are reflected in the median values of HEI and AHEI scores in this population (HEI:34 and AHEI: 40) which were lower than the values reported in the United States (HEI ≈ 59 and AHEI ≈ 64), and Europe (HEI ≈ 50–64 across different countries).^51–55^ However, the median values of AMED and DASH scores in this population were comparable to those from high-income countries.^51–53,55^ These results might indicate dietary indices that use population ranking rather than absolute cutoffs for assigning values for consumption of dietary components might be more applicable to LMICs and could provide for better estimation of individual diet quality and its association with different health outcomes in the developing regions. Our previous studies that assessed the association of diet with risk of all-cause and cause-specific mortality,^9^ and lung cancer incidence also showed more consistent results for ranking-based scores (DAHS and AMED) compared to cutoff-based scores (HEI and AHEI).

 While AMED, DASH, and WCRF-AICR scores showed more consistent associations with UGI cancers in this population, these associations varied by cancer type. These variations could be due to the differences in components and weight of individual dietary factors in each score. For example, AMED assigns values of 0 and 1 for each component based on its consumption amount being below or above the median value for the population, and it does not consider the intake of salt, sugar, and dairy products; DASH assigns 0 to 5 values for each component based on its consumption amount being within the quantiles of consumption in the population, and it does not consider fatty acids, alcohol, or fibers; WCRF-AICR assigns values of 0 and 1 for each component based on specific cutoffs that were created based on cancer studies in different populations and is the only score that includes energy density and fiber components. Current evidence indicates that the effects of different dietary components on cancers along the GI tract might vary based on site and even histology of GI cancers.^8^

 The main strengths of this study are its large sample size, long follow-up duration with minimum loss ( < 1%), and using validated baseline questionnaires for the assessment of diet and other exposures. One limitation in this study is using self-reported FFQ data only at baseline to classify the main exposure which might have resulted in misclassifications of people due to inaccurate responses and the possibility of changing dietary behavior during the follow-up. However, the prospective nature of this study and the good validity and reliability of the questionnaires, that was evident during a 12-month period of repeated testing, are likely to have minimized the possibility of misclassifications of exposure. Like any observational studies, residual confounding, particularly related to healthier lifestyle in those with healthier diets, remains a concern. However, we tried to address this potential bias by tight adjustments for potential confounders and performing various stratified analyses. Reverse causality is another potential bias that we tried to address by repeating the analysis after dropping the first two years of follow-up that showed results comparable to the main analysis.

## Supplementary Files

Supplementary file 1 contains File S1 and Tables S1-S7.
