# Supplementary material for: Diet Quality and Subsequent Incidence of Upper Gastrointestinal Cancers: Results from the Golestan Cohort Study
Source: Arch Iran Med. 2023 Sep 1;26(9):489–98. doi: 10.34172/aim.2023.74 (PMC10862059; doi:10.34172/aim.2023.74)
Supplement: Supplementary file 1 — contains File S1 and Tables S1-S7. [file aim-26-489-s001.pdf]

Arch Iran Med. September 2023;26(9):489-S1.

10.34172/aim.2023.74

File S1

Validity and reliability of the questionnaires

The details of validation studies have been published previously [1–3]. Briefly, in the pilot phase of the study, >1000 individuals were interviewed, and two months later, a repeat interview was performed for 131 participants which showed good agreement with the first interview [1]. The validity of the general questionnaire data regarding the use of opium and tobacco was assessed in a subgroup of participants by comparing their questionnaire responses with the presence of codeine or morphine (for opium) and cotinine (for tobacco) in their urine, which showed good sensitivity and specificity for the questionnaire to detect current opium and tobacco use [1,3].

To validate the FFQ, twelve 24-hour recall dietary questionnaires (1 per month) and four FFQs (1 per season) were administered during one year to 131 participants [2]. Furthermore, four 24-hour urine samples were collected from these participants and their responses were compared with the urinary excretion of selected nutrients [2]. The results showed good correlations between FFQ and recall data on food group and nutrient intakes, and acceptable correlations between FFQ data and urinary biomarker measurements [2].

Full details of calculating the dietary scores

The Healthy Eating Index 2015 (HEI-2015), Alternative Healthy Eating Index 2010 (AHEI-2010), Alternative Mediterranean Diet (AMED), Dietary Approaches to Stop Hypertension (DASH-Fung), and World Cancer Research Fund – American Institute for Cancer Research (WCRF-AICR) were calculated by Hashemian *et al.* for the participants of the Golestan Cohort Study [4]. To create components of the HEI-2015 and AHEI-2010 scores, we converted the daily intakes from grams to cup and ounce equivalents using the Food Patterns Equivalents Database (FPED) 2013–14 [5]. The FPED units are ounce and cup equivalents and can be converted to standard units as follows: 1 ounce=28.35 g and 1 cup= 225 mL. For fruits and vegetables, we used an extensive list of one cup equivalent weights for fruits and vegetables in the FPED [5]. For example, for canned fruit in light syrup, 65% fruit was assumed. For grain products such as bread, dough and cake, made with flour, each 16 grams of flour present in a food was used as the basis for defining a one-ounce grain equivalent, the rationale being that one standard slice of bread has been defined as equal to one-ounce grain equivalent, which will contain 16 grams of flour [5]. For intact grains such as rice and pasta, cooked grains were converted to the uncooked forms with conversion factors 0.36 and 0.37, respectively [6], and one-ounce equivalent of grains was defined as 28.35 grams [5]. In the FFQ, multi-ingredient foods such as pizza were not queried, so we did not have to disaggregate the foods. However, protein foods were further disaggregated to lean fraction and fat as follows: meat and poultry were disaggregated to lean meat and solid fat fractions; and seafood and nuts were disaggregated to lean protein and oil fractions. Similarly, dairy foods were further disaggregated to a low fat dairy fraction, similar to skim milk, and a solid fat fraction [5].

Food items were assigned to food groups according to Table S1. The dietary scores were calculated as follows and were categorized into quartiles. Note that the numbers of participants were not always equal in each quartile.

HEI-2015

The HEI-2015 includes 13 components for a total of 100 points based on the 2015 Dietary Guidelines for Americans [7], including 9 adequacy components: total fruit (including fruit juice); whole fruit; total vegetables; greens and beans (including dark green vegetables and legumes); whole grains; dairy; total protein foods [includes meat and poultry (lean fraction), eggs, seafood, nuts, seeds, soy and legumes]; seafood and plant proteins [including seafood, nuts, seeds, soy and legumes]; fatty acids [ratio of polyunsaturated fatty acids (PUFAs) and monounsaturated fatty acids (MUFAs) to saturated fatty acids (SFAs)] (Table S2); and four moderation components: SFAs, refined grains, sodium, and added sugars (Table S2). The components were calculated per 1000 kcal/d (energy density model).

AHEI-2010

The AHEI-2010 includes 11 components for a total of 110 points [8–10]. The AHEI-2010 includes fruits, vegetables, whole grains, red and processed meat, nuts and legumes, trans fats, omega-3 fatty acids, PUFAs, sugary sweetened beverages (SSB) and fruit juice, sodium, and alcohol (Table S2). The AHEI-2010 is similar to HEI-2015; however, potatoes are not included in the vegetable group in this score. Also, the AHEI-2010 uses an absolute intake method instead of a nutrient density method [9]. SSBs were defined as any beverages containing a caloric sweetener, even if added after purchase [11]. Therefore, we included sweet tea if it contained approximately one half (or more) of the sugar and calories of regular sodas [11].

AMED

The AMED includes nine components for a total of nine points, based on the Mediterranean diet [12]: all vegetables (excluding potatoes), all fruits (including juice), nuts, legumes, fish, whole grains, MUFA to SFA ratio, red and processed meat, and alcohol. We applied one point where reported red and processed meat consumption was less than the sex-specific median. For other components, intakes above the sex-specific median of the study subjects received one point. All other intakes received 0 points (Table S2).

DASH-Fung score

The DASH score created by Fung includes eight components for a total of 40 points: seven food groups and one nutrient [13]. Scores are based on sex-specific quintiles in the population. The highest quintile of intake for fruits, vegetables, low-fat dairy, whole grains, nuts and legumes each received five points, and the highest quintile of intake for red and processed meat, SSBs, and sodium each received one point (Table S2).

WCRF-AICR score

The WCRF/AICR score includes seven dietary components: energy dense foods which cause weight gain [14], fruits and vegetables, red and processed meat, alcohol, sugary drinks, fiber, and sodium; plus nondietary components associated with cancer risk including physical activity, body fatness, and breastfeeding [15]. However, we only calculated the dietary components in this study, to allow for comparability with the other diet-only scores. Energy density was calculated as energy from all solid and semi-solid foods divided by the weights (g) of these foods. Drinks (including water, tea, green tea, juice, soft drinks, alcoholic drinks and milk) were not included in the calculation of energy density [14]. For each component, participants who met the official recommendation received one point, those who met an intermediate recommendation received one-half of a point, and those who met neither recommendation received zero points [15] (Table S2).

Alcohol was queried on the demographic baseline questionnaire. Since alcohol intake is not common in this population (4%), all participants received a zero for it in each dietary score. Also, no item in the FFQ was whole-grain, because they are not consumed by this population, so all respondents received a zero for whole grains. We did not modify the scores and did not delete these components, so that we could compare the scores from this population with others.

References

1. Pourshams A, Saadatian-Elahi M, Nouraie M, Fazeltabar Malekshah A, Rakhshani N, Salahi R, et al. Golestan cohort study of oesophageal cancer: feasibility and first results. Br J Cancer. 2005;92(1):176-81. doi: 10.1038/sj.bjc.6602249.

2. Fazeltabar Malekshah A, Kimiagar M, Saadatian-Elahi M, Pourshams A, Nouraie M, Goglani G, et al. Validity and reliability of a new food frequency questionnaire compared to 24 h recalls and biochemical measurements: pilot phase of Golestan cohort study of esophageal cancer. Eur J Clin Nutr. 2006;60(8):971-7. doi: 10.1038/sj.ejcn.1602407.

3. Abnet CC, Saadatian-Elahi M, Pourshams A, Boffetta P, Feizzadeh A, Brennan P, et al. Reliability and validity of opiate use self-report in a population at high risk for esophageal cancer in Golestan, Iran. Cancer Epidemiol Biomarkers Prev. 2004;13(6):1068-70.

4. Hashemian M, Farvid MS, Poustchi H, Murphy G, Etemadi A, Hekmatdoost A, et al. The application of six dietary scores to a Middle Eastern population: a comparative analysis of mortality in a prospective study. Eur J Epidemiol. 2019;34(4):371-82. doi: 10.1007/s10654-019-00508-3.

5. Bowman SA, Clemens JC, Friday JE, Lynch KL, Moshfegh AJ. Food Patterns Equivalents Database 2013-14: Methodology and User Guide. Food Surveys Research Group, Beltsville Human Nutrition Research Center, Agricultural Research Service, U.S. Department of Agriculture, Beltsville, Maryland [Internet]. Available from: http://www.ars.usda.gov/nea/bhnrc/fsrg. Accessed September 20, 2019. Beltsville, MD: Food Surveys Research Group; 2017.

6. Bowman SA, Martin CL, Carlson JL, Clemens JC, Lin BH, Moshfegh AJ. Food Intakes Converted to Retail Commodities Databases: 2003-08: Methodology and User Guide. A.R.S. U.S. Department of Agriculture, Beltsville, MD, and U.S. Department of Agriculture, Economic Research Service, Editor. [Internet]. Washington, DC. p. 48.; 2013. Available from: https://data.nal.usda.gov/dataset/food-intakes-converted-retail-commodities-databases-ficrcd. Accessed September 20, 2019. 7. National Cancer Institute; National Institutes of Health. Dietary Patterns Methods Project [Internet]. Available from: https://epi.grants.cancer.gov/hei/hei-2015-table1.html.

8. Reedy J, Krebs-Smith SM, Miller PE, Liese AD, Kahle LL, Park Y, et al. Higher diet quality is associated with decreased risk of all-cause, cardiovascular disease, and cancer mortality among older adults. J Nutr. 2014;144(6):881-9. doi: 10.3945/jn.113.189407.

9. McCullough ML, Feskanich D, Stampfer MJ, Giovannucci EL, Rimm EB, Hu FB, et al. Diet quality and major chronic disease risk in men and women: moving toward improved dietary guidance. Am J Clin Nutr. 2002;76(6):1261-71. doi: 10.1093/ajcn/76.6.1261.

10. Chiuve SE, Fung TT, Rimm EB, Hu FB, McCullough ML, Wang M, et al. Alternative dietary indices both strongly predict risk of chronic disease. J Nutr. 2012;142(6):1009-18. doi: 10.3945/jn.111.157222.

11. Miller PE, McKinnon RA, Krebs-Smith SM, Subar AF, Chriqui J, Kahle L, et al. Sugar-sweetened beverage consumption in the U.S.: novel assessment methodology. Am J Prev Med. 2013;45(4):416-21. doi: 10.1016/j.amepre.2013.05.014.

12. Fung TT, McCullough ML, Newby PK, Manson JE, Meigs JB, Rifai N, et al. Diet-quality scores and plasma concentrations of markers of inflammation and endothelial dysfunction. Am J Clin Nutr. 2005;82(1):163-73. doi: 10.1093/ajcn.82.1.163.

13. Fung TT, Chiuve SE, McCullough ML, Rexrode KM, Logroscino G, Hu FB. Adherence to a DASH-style diet and risk of coronary heart disease and stroke in women. Arch Intern Med. 2008;168(7):713-20. doi: 10.1001/archinte.168.7.713.

14. Muller DC, Murphy N, Johansson M, Ferrari P, Tsilidis KK, Boutron-Ruault MC, et al. Modifiable causes of premature death in middle-age in Western Europe: results from the EPIC cohort study. BMC Med. 2016;14:87. doi: 10.1186/s12916-016-0630-6.

15. Romaguera D, Vergnaud AC, Peeters PH, van Gils CH, Chan DS, Ferrari P, et al. Is concordance with World Cancer Research Fund/American Institute for Cancer Research guidelines for cancer prevention related to subsequent risk of cancer? Results from the EPIC study. Am J Clin Nutr. 2012;96(1):150-63. doi: 10.3945/ajcn.111.031674.

Table S1. Assigning foods queried in the food frequency questionnaire of the Golestan Cohort Study to food groups

**Food groups**	**Food items included**
Total fruits	All fresh fruits, dried fruit, cooked fruit, lemon juice, orange juice
Whole fruits	Total fruits excluding juice
Vegetables [1]	Dark green, red and orange, starchy, and other vegetables[1]
Whole grains	None of the food items in the FFQ considered whole grains
Refined grains	Rice, all kinds of bread, flour, dough, noodle, pasta
Dairy	Pasteurized milk, raw milk, pasteurized cheese, non-pasteurized cheese, low/medium-fat yogurt, high fat yogurt (homemade), Greek yogurt, yogurt drink, camel yogurt drink, pasteurized dried yogurt paste (*Kashk*), dried *Kashk*, *Agharan* (a local product made from camel milk)
Low fat dairy	Pasteurized milk (≤2.5% fat), pasteurized cheese, low/medium-fat yogurt (≤2.5% fat), yogurt drink (*doogh*), camel yogurt drink, pasteurized dried yogurt paste (*Kashk*), dried *Kashk*
Total Protein Foods (AHEI[2])	Total meat (including organ meats and cured meats); poultry; seafood; eggs; nuts and seeds; soy; and beans and peas
Seafood and Plant Proteins	Seafoods, nuts, seeds, soy products, and beans and peas
Greens and Beans	Dark green vegetables, and any beans and peas (legumes)
Red/processed meat	Unprocessed red meat (beef or lamb, hamburger), liver, chicken liver; and processed red meat (sausage)
Nuts	Walnuts, peanuts, mixed nuts
Legumes	White bean, red bean, pinto bean, chickpea, split pea, soy bean, and lentil
Fish	Stellate sturgeon, Carp, Smoked fish, Salted fish, and tuna
Sugary sweetened beverages	Soft drink, Commercial juice, sweet beverage, sweet tea

Table footnote

[1] including potato for HEI and excluding potato for other scores

[2] Alternative Healthy Eating Index (AHEI)

Table S2. Components and minimum and maximum criteria for dietary scores

	HEI			AHEI			AMED			DASH			WCRF/AICR		
**Groups/ Nutrients**	Items (n=13)	Criteria		Items (n=11)	Criteria		Items (n=9)	Criteria		Items (n=8)	Criteria		Items (n=7)	Criteria	
		Min (0)	Max(10)		Min (0)	Max (10)		Min (0)	Max (1)		Min (1)	Max (5)		Min (0)	Max (1)
**Fruits**	Total fruits, c/k	0	≥0.8	Fruits, c/d	0	≥2	Fruits	<m	≥m	Fruits	Q1	Q5	Fruits & Vegetables, g/d	<200	≥400
	Whole fruits, c/k	0	≥0.4												
**Vegetables**	Total vegetables[1], c/k	0	≥1.1	Vegetables, c/d	0	≥2.5	Vegetables	<m	≥m	Vegetables	Q1	Q5			
	Greens & beans[2], c/k	0	≥0.2												
**Grains**	Whole grains, oz/k	0	≥1.5	Whole grains, oz/d (MALE, FEMALE)	0	≥6 & ≥5	Whole grains	<m	≥m	Whole grains	Q1	Q5			
	Refined grains, oz/k	≥4.3	≤1.8												
**Dairy**	Dairy, c/k	0	≥1.3							Low fat dairy	Q1	Q5			
**Protein foods**	Total protein foods[3], oz/k	0	≥2.5	Red/ processed meat, oz/d	≥1.5	0	Red/ processed meat	<m	≥m	Red/ processed meat	Q5	Q1	Red, g/w & processed meat, g/d	≥500 & ≥ 50	<500 & < 3
	Seafood, plant protein[4],oz/k	0	≥0.8	Nuts, soy, legumes, oz/d	0	≥1	Nuts	<m	≥m	Nuts, legumes	Q1	Q5			
							Legumes	<m	≥m						
							Fish	<m	≥m						
**Fat**	(PUFA+MUFA) /SFAs	≤1.2	≥2.5	PUFA, %E	≤2	≥10	MUFA/ SFA	<m	≥m				Energy dense foods, kcal/100g/d	≥175	<125
	SFAs, %E	≥16	≤8	Omega-3, mg/d	0	250									
				Trans fat, % E	≥4	≤0.5									
**Sodium**	Sodium, g/k	≥2	≤1.1	Sodium	Highest decile	Lowest decile				Sodium	Q5	Q1	Sodium, g/d	≥4.8	<2.4
**Sugars**	Added sugars, %E	≥26	≤6.5	SSB, fruit juice, c/d	≥1	0				SSB	Q5	Q1	Sugary drinks, g/d	>250	0
**Alcohol**				Alcohol (d/d)(M,F)	≥3.5 &≥2.5	0.5-2 &0.5-1.5	Alcohol	<m	≥m				Alcohol, g/d (MALE, FEMALE)	>30 & >20	≤20 & ≤10
**Fiber**													Fiber, g/d	<12.5	≥25

Table footnote

Healthy Eating Index (HEI), cup equivalent /1000 kcal (c/k), ounce equivalent /1000 kcal (oz/k), Polyunsaturated Fatty Acids (PUFA), Monounsaturated Fatty Acids (MUFA), Saturated Fatty Acids (SFA), Energy (E), grams per 1000 kcal (g/k); Alternative Healthy Eating Index (AHEI), cup equivalent/ day (c/d), ounce equivalent /day (oz/d), male and female, respectively (M,F), milligrams/day (mg/d), sugary sweetened beverages (SSB), drinks/day (d/d),; Alternate Mediterranean Diet (AMED), Median (m); Dietary Approaches to Stop Hypertension (DASH); World Cancer Research Fund/American Institute for Cancer Research index (WCRF/AICR), grams/week (g/w), grams/day (g/d); DASH created by Mellen (DASH-Mellen), milligrams per 1000 kcal (mg/k), Magnesium (Mg), Calcium (Ca), Potassium (K)

1 Including potatoes

2 Dark green vegetables and legumes

3 Total meat (including organ meats and cured meats), poultry, seafood; eggs; nuts and seeds; soy; legumes

4 Seafood; nuts and seeds; soy; legumes (beans and peas)

Table S3. Sex-stratified analysis to assess the association between dietary scores and incident upper gastrointestinal cancers in the Golestan Cohort Study.

	Male Subgroup			Female subgroup		
	**Cancer cases**	**Q4 vs. Q1**	**Trend p-value**	**Cancer cases**	**Q4 vs. Q1**	**Trend p-value**
**Esophageal Cancer**	187			172		
**HEI**						
N of participants		4,798 vs. 5,799	-		6,693 vs. 7,480	-
HR (95% CI) [1]		0.72 (0.44 – 1.17)	0.14		1.08 (0.70 – 1.67)	0.58
**AHEI**						
N of participants		5,032 vs. 5,505	-		6,237 vs. 7,139	-
HR (95% CI) [1]		0.88 (0.56 – 1.37)	0.41		0.94 (0.60 – 1.45)	0.44
**AMED**						
N of participants		5,553 vs. 5,677	-		7,663 vs. 7,803	-
HR (95% CI) [1]		0.69 (0.43 – 1.13)	0.09		0.70 (0.42 – 1.15)	0.22
**DASH**						
N of participants		6,762 vs. 4,940	-		9,089 vs. 6,669	-
HR (95% CI) [1]		0.80 (0.54 – 1.18)	0.16		0.89 (0.59 – 1.34)	0.54
**WCRF-AICR**						
N of participants		6,473 vs. 4,744	-		5,838 vs. 9,195	-
HR (95% CI) [1]		0.69 (0.44 – 1.10)	0.07		1.47 (0.87 – 2.50)	0.13
**Stomach Cancer**	259			99		
**HEI**						
N of participants		4,798 vs. 5,799	-		6,693 vs. 7,480	-
HR (95% CI) [1]		0.92 (0.63 – 1.34)	0.87		0.85 (0.47 – 1.54)	0.74
**AHEI**						
N of participants		5,032 vs. 5,505	-		6,237 vs. 7,139	-
HR (95% CI) [1]		0.89 (0.62 – 1.28)	0.87		0.61 (0.32 – 1.18)	0.07
**AMED**						
N of participants		5,553 vs. 5,677	-		7,663 vs. 7,803	-
HR (95% CI) [1]		0.98 (0.66 – 1.44)	0.94		0.73 (0.40 – 1.33)	0.20
**DASH**						
N of participants		6,762 vs. 4,940	-		9,089 vs. 6,669	-
HR (95% CI) [1]		**0.66 (0.47 – 0.93)**	**0.03**		0.90 (0.52 – 1.54)	0.6
**WCRF-AICR**						
N of participants		6,473 vs. 4,744	-		5,838 vs. 9,195	-
HR (95% CI) [1]		**0.63 (0.42 – 0.94)**	**0.03**		**0.47 (0.22 – 0.99)**	**0.03**

Table footnote

N: number; **Q:** quartile; **HEI:** Healthy Eating Index 2015; **AHEI:** Alternative Healthy Eating Index 2010; **AMED:** Alternate Mediterranean Diet; **DASH:** Dietary Approaches to Stop Hypertension; **WCRF/AICR:** World Cancer Research Fund/American Institute for Cancer Research index

[1] Models are adjusted for residence district, socioeconomic status, ethnicity, education, BMI, physical activity level, cumulative cigarettes smoked, cumulative opium consumed, alcohol consumption, and energy intake.

Table S4. Socioeconomic-stratified analysis to assess the association between dietary scores and incident upper gastrointestinal cancers in the Golestan Cohort Study.

	Wealth score lower than the median			Wealth score higher than the median		
	**Cancer cases**	**Q4 vs. Q1**	**Trend p-value**	**Cancer cases**	**Q4 vs. Q1**	**Trend p-value**
**Esophageal Cancer**	243			116		
**HEI**						
N of participants		3,494 vs. 8,803	-		7,997 vs. 4,476	-
HR (95% CI) [1]		1.18 (0.81 – 1.70)	0.64		0.60 (0.33 – 1.06)	0.06
**AHEI**						
N of participants		4,187 vs. 7,197	-		7,082 vs. 5,447	-
HR (95% CI) [1]		0.98 (0.68 – 1.43)	0.41		0.87 (0.50 – 1.52)	0.63
**AMED**						
N of participants		3,988 vs. 8,914	-		9,228 vs. 4,566	-
HR (95% CI) [1]		0.73 (0.47 – 1.12)	0.08		0.75 (0.42 – 1.33)	0.34
**DASH**						
N of participants		5,871 vs. 7,125	-		9,980 vs. 4,484	-
HR (95% CI) [1]		1.10 (0.78 – 1.54)	0.89		**0.52 (0.31 – 0.85)**	**0.01**
**WCRF-AICR**						
N of participants		3,955 vs. 9,194	-		8,356 vs. 4,745	-
HR (95% CI) [1]		1.08 (0.70 – 1.66)	0.86		0.81 (0.46 – 1.44)	0.52
**Stomach Cancer**	211			147		
**HEI**						
N of participants		3,494 vs. 8,803	-		7,997 vs. 4,476	-
HR (95% CI) [1]		1.18 (0.81 – 1.70)	0.64		0.60 (0.33 – 1.06)	0.06
**AHEI**						
N of participants		4,187 vs. 7,197	-		7,082 vs. 5,447	-
HR (95% CI) [1]		0.68 (0.44 – 1.06)	0.24		0.98 (0.61 – 1.58)	0.72
**AMED**						
N of participants		3,988 vs. 8,914	-		9,228 vs. 4,566	-
HR (95% CI) [1]		0.81 (0.51 – 1.28)	0.18		0.96 (0.59 – 1.57)	0.79
**DASH**						
N of participants		5,871 vs. 7,125	-		9,980 vs. 4,484	-
HR (95% CI) [1]		**0.65 (0.45 – 0.95)**	**0.02**		0.83 (0.52 – 1.33)	0.45
**WCRF-AICR**						
N of participants		3,955 vs. 9,194	-		8,356 vs. 4,745	-
HR (95% CI) [1]		**0.55 (0.34 – 0.89)**	**0.02**		**0.56 (0.33 – 0.94)**	**0.02**

<u>**Table footnote**</u>

N: number; **Q:** quartile; **HEI:** Healthy Eating Index 2015; **AHEI:** Alternative Healthy Eating Index 2010; **AMED:** Alternate Mediterranean Diet; **DASH:** Dietary Approaches to Stop Hypertension; **WCRF/AICR:** World Cancer Research Fund/American Institute for Cancer Research index

[1] Models are adjusted for sex, residence district, ethnicity, education, BMI, physical activity level, cumulative cigarettes smoked, cumulative opium consumed, alcohol consumption, and energy intake.

Table S5. BMI-stratified analysis to assess the association between dietary scores and incident upper gastrointestinal cancers in the Golestan Cohort Study.

	**BMI ≤ 25 at recruitment**			**BMI > 25 at recruitment**		
	**Cancer cases**	**Q4 vs. Q1**	**Trend p-value**	**Cancer cases**	**Q4 vs. Q1**	**Trend p-value**
**Esophageal Cancer**	224			135		
**HEI**						
N of participants		3,381 vs. 6,776	-		8,110 vs. 6,503	-
HR (95% CI) [1]		0.87 (0.57 – 1.32)	0.43		0.93 (0.56 – 1.53)	0.76
**AHEI**						
N of participants		3,808 vs. 5,727	-		7,461 vs. 6,917	-
HR (95% CI) [1]		1.05 (0.71 – 1.55)	0.57		0.73 (0.44 – 1.21)	0.26
**AMED**						
N of participants		6,738 vs. 3,969	-		6,742 vs. 9,247	-
HR (95% CI) [1]		**0.56 (0.34 – 0.91)**	**0.04**		0.84 (0.50 – 1.40)	0.35
**DASH**						
N of participants		5,462 vs. 5,388	-		10,389 vs. 6,221	-
HR (95% CI) [1]		0.92 (0.64 – 1.30)	0.35		0.72 (0.45 – 1.15)	0.26
**WCRF-AICR**						
N of participants		3,842 vs. 6,889	-		8,469 vs. 6,809	-
HR (95% CI) [1]		0.93 (0.60 – 1.46)	0.78		1.08 (0.61 – 1.89)	0.91
**Stomach Cancer**	177			181		
**HEI**						
N of participants		3,381 vs. 6,776	-		8,110 vs. 6,503	-
HR (95% CI) [1]		0.93 (0.58 – 1.48)	0.75		0.90 (0.58 – 1.39)	0.96
**AHEI**						
N of participants		3,808 vs. 5,727	-		7,461 vs. 6,917	-
HR (95% CI) [1]		0.74 (0.47 – 1.16)	0.30		0.92 (0.59 – 1.45)	0.66
**AMED**						
N of participants		6,738 vs. 3,969	-		6,742 vs. 9,247	-
HR (95% CI) [1]		1.06 (0.66 – 1.70)	0.74		0.80 (0.51 – 1.24)	0.27
**DASH**						
N of participants		5,462 vs. 5,388	-		10,389 vs. 6,221	-
HR (95% CI) [1]		0.73 (0.48 – 1.10)	0.23		0.71 (0.47 – 1.07)	0.09
**WCRF-AICR**						
N of participants		3,842 vs. 6,889	-		8,469 vs. 6,809	-
HR (95% CI) [1]		**0.49 (0.29 – 0.83)**	**0.01**		0.68 (0.42 – 1.10)	0.09

Table footnote

N: number; **Q:** quartile; **HEI:** Healthy Eating Index 2015; **AHEI:** Alternative Healthy Eating Index 2010; **AMED:** Alternate Mediterranean Diet; **DASH:** Dietary Approaches to Stop Hypertension; **WCRF/AICR:** World Cancer Research Fund/American Institute for Cancer Research index

[1] Models are adjusted for sex, residence district, socioeconomic status, ethnicity, education, physical activity level, cumulative cigarettes smoked, cumulative opium consumed, alcohol consumption, and energy intake.

Table S6. Association between different dietary scores and incidence of gastrointestinal cancers in the Golestan Cohort Study **after dropping patients who did not have histologic confirmation for cancer diagnosis.**

	Cancer cases	Q4 vs. Q1	Trend p-value
**Esophageal Cancer**	309		
**HEI**			
N of participants		11,458 VS. 13,217	
HR (95% CI) [1]		0.95 (0.68 – 1.34)	0.72
**AHEI**			
N of participants		11,232 VS. 12,587	
HR (95% CI) [1]		0.93 (0.66 – 1.30)	0.39
**AMED**			
N of participants		13,183 vs. 13,414	
HR (95% CI) [1]		**0.71 (0.49 – 1.02)**	**0.03**
**DASH**			
N of participants		15,798 vs. 11,556	
HR (95% CI) [1]		0.79 (0.58 – 1.07)	0.08
**WCRF-AICR**			
N of participants		12,272 vs. 13,873	
HR (95% CI) [1]		0.93 (0.63 – 1.37)	0.71
**Stomach Cancer**	278		
**HEI**			
N of participants		11,458 VS. 13,217	
HR (95% CI) [1]		0.90 (0.63 – 1.28)	0.71
**AHEI**			
N of participants		11,232 VS. 12,587	
HR (95% CI) [1]		0.77 (0.54 – 1.10)	0.12
**AMED**			
N of participants		13,183 vs. 13,414	
HR (95% CI) [1]		0.93 (0.65 – 1.35)	0.62
**DASH**			
N of participants		15,798 vs. 11,556	
HR (95% CI) [1]		**0.70 (0.50 – 0.96)**	**0.05**
**WCRF-AICR**			
N of participants		12,272 vs. 13,873	
HR (95% CI) [1]		**0.63 (0.43 – 0.94)**	**0.02**

Table footnote

N: number; **Q:** quartile; **HEI:** Healthy Eating Index 2015; **AHEI:** Alternative Healthy Eating Index 2010; **AMED:** Alternate Mediterranean Diet; **DASH:** Dietary Approaches to Stop Hypertension; **WCRF/AICR:** World Cancer Research Fund/American Institute for Cancer Research index

[1] Models are adjusted for sex, residence district, socioeconomic status, ethnicity, education, BMI, physical activity level, cumulative cigarettes smoked, cumulative opium consumed, alcohol consumption, and energy intake.

Table S7. Association between different dietary scores and incidence of gastrointestinal cancers in the Golestan Cohort Study **after dropping the first two years of follow-up.**

	Cancer cases	Q4 vs. Q1	Trend p-value
**Esophageal Cancer**	281		
**HEI**			
N of participants		11,329 vs. 13,069	
HR (95% CI) [1]		0.99 (0.69 – 1.40)	0.77
**AHEI**			
N of participants		11,101 vs. 12,455	
HR (95% CI) [1]		0.96 (0.68 – 1.37)	0.52
**AMED**			
N of participants		13,078 vs. 13,236	
HR (95% CI) [1]		0.76 (0.51 – 1.11)	0.15
**DASH**			
N of participants		15,645 vs. 11,432	
HR (95% CI) [1]		0.85 (0.62 – 1.18)	0.33
**WCRF-AICR**			
N of participants		12,171 vs. 13,709	
HR (95% CI) [1]		1.13 (0.77 – 1.65)	0.72
**Stomach Cancer**	308		
**HEI**			
N of participants		11,329 vs. 13,069	
HR (95% CI) [1]		0.87 (0.62 – 1.22)	0.55
**AHEI**			
N of participants		11,101 vs. 12,455	
HR (95% CI) [1]		0.79 (0.56 – 1.11)	0.18
**AMED**			
N of participants		13,078 vs. 13,236	
HR (95% CI) [1]		0.88 (0.62 – 1.25)	0.52
**DASH**			
N of participants		15,645 vs. 11,432	
HR (95% CI) [1]		**0.66 (0.48 – 0.91)**	**0.01**
**WCRF-AICR**			
N of participants		12,171 vs. 13,709	
HR (95% CI) [1]		**0.59 (0.41 – 0.87)**	**0.01**

Table footnote

N: number; **Q:** quartile; **HEI:** Healthy Eating Index 2015; **AHEI:** Alternative Healthy Eating Index 2010; **AMED:** Alternate Mediterranean Diet; **DASH:** Dietary Approaches to Stop Hypertension; **WCRF/AICR:** World Cancer Research Fund/American Institute for Cancer Research index

[1] Models are adjusted for sex, residence district, socioeconomic status, ethnicity, education, BMI, physical activity level, cumulative cigarettes smoked, cumulative opium consumed, alcohol consumption, and energy intake.
